# Application of an Inclined Settler for Cell Culture-Based Influenza A Virus Production in Perfusion Mode

**DOI:** 10.3389/fbioe.2020.00672

**Published:** 2020-07-02

**Authors:** Juliana Coronel, Gwendal Gränicher, Volker Sandig, Thomas Noll, Yvonne Genzel, Udo Reichl

**Affiliations:** ^1^Max Planck Institute for Dynamics of Complex Technical Systems, Magdeburg, Germany; ^2^ProBioGen AG, Berlin, Germany; ^3^Institute of Cell Culture Technology, Bielefeld University, Bielefeld, Germany; ^4^Bioprocess Engineering, Otto von Guericke University Magdeburg, Magdeburg, Germany

**Keywords:** inclined settler, influenza vaccine, perfusion, suspension cell culture, continuous harvesting

## Abstract

Influenza viruses have been successfully propagated using a variety of animal cell lines in batch, fed-batch, and perfusion culture. For suspension cells, most studies reported on membrane-based cell retention devices typically leading to an accumulation of viruses in the bioreactor in perfusion mode. Aiming at continuous virus harvesting for improved productivities, an inclined settler was evaluated for influenza A virus (IAV) production using the avian suspension cell line AGE1.CR.pIX. Inclined settlers present many advantages as they are scalable, robust, and comply with cGMP regulations, e.g., for recombinant protein manufacturing. Perfusion rates up to 3000 L/day have been reported. In our study, successful growth of AGE1.CR.pIX cells up to 50 × 10^6^ cells/mL and a cell retention efficiency exceeding 96% were obtained with the settler cooled to room temperature. No virus retention was observed. A total of 5.4–6.5 × 10^13^ virions were produced while a control experiment with an ATF system equaled to 1.9 × 10^13^ virions. For infection at 25 × 10^6^ cells/mL, cell-specific virus yields up to 3474 virions/cell were obtained, about 5-fold higher than for an ATF based cultivation performed as a control (723 virions/cell). Trypsin activity was shown to have a large impact on cell growth dynamics after infection following the cell retention device, especially at a cell concentration of 50 × 10^6^ cells/mL. Further control experiments performed with an acoustic settler showed that virus production was improved with a heat exchanger of the inclined settler operated at 27°C. In summary, cell culture-based production of viruses in perfusion mode with an inclined settler and continuous harvesting can drastically increase IAV yields and possibly the yield of other viruses. To our knowledge, this is the first report to show the potential of this device for viral vaccine production.

## Introduction

While most commercial influenza vaccines are produced on chicken eggs (Barr et al., 2018), various animal cell-based vaccines have also been licensed (Milian and Kamen, 2015), or are under development (Krammer and Palese, 2015). Manufacturing costs of cell-based vaccines tend to be higher than for egg-based products. However, it has been argued that costs can be decreased significantly using highly optimized cell culture processes (Barr et al., 2018). One approach is bioreactor operation in perfusion mode to support growth to high cell concentration associated with an efficient virus production phase. For this purpose, suspension cells can be cultivated in bioreactor systems coupled to cell retention devices (Woodside et al., 1998; Genzel et al., 2014; Tapia et al., 2016).

Operation in perfusion mode is generally more efficient and more flexible than other cultivation strategies allowing high cell concentrations (10^7^–10^8^ cells/mL) and high volumetric productivities (Walther et al., 2015; Bielser et al., 2018; Chen et al., 2018). In addition, product quality attributes can be improved in perfusion cultures, for example, by reducing product heterogeneity (Bielser et al., 2018), or preventing the accumulation of growth inhibitors and metabolic waste products. Most perfusion studies in lab-scale bioreactors for virus vaccine production were carried out using filtration systems, such as spin-filters (Perrin et al., 1995), tangential flow filtration (TFF; Nikolay et al., 2018; Coronel et al., 2019a), or alternating tangential flow filtration (ATF; Genzel et al., 2014; Vazquez-Ramirez et al., 2018; Gränicher et al., 2019). The latter is the most commonly used perfusion system in recombinant protein production (Bielser et al., 2018). In our experience, perfusion with hollow-fiber membranes [e.g., 0.2 μm pore size made of polyethersulfone (PES)] did not support efficient virus harvesting. Virus retention inside the bioreactor was observed using various setups, including other membrane materials and larger pore sizes, for different viruses, such as influenza A virus (IAV; Genzel et al., 2014), modified vaccinia Ankara (MVA) virus (Vazquez-Ramirez et al., 2018), and flaviviruses (Nikolay et al., 2018). This is not only due to the relatively large size of some viruses (>80 nm for influenza virus) and possible virus aggregate formation but also depends on virus-induced apoptosis and cell lysis, and the release of host cell DNA and proteins. This potentially results in cake formation, narrows pores or blocks completely the membranes and thus prevents continuous virus harvesting through the membrane. Examples of cell retention devices that potentially enable continuous virus harvesting are: acoustic settler (Petiot et al., 2011), centrifuge, hydrocyclone (Elsayed et al., 2006), and inclined settler (Woodside et al., 1998; Castilho, 2014). However, few studies applying these technologies for virus vaccine production were reported. For example, Petiot et al. (2011) reported on an acoustic cell retention system for IAV production in HEK293 cells. Degradation of viral particles in the bioreactor was reduced when production was carried out under mild hypothermia (35°C) and the supernatant was immediately stored at 2–8°C (Petiot et al., 2011).

**FIGURE 1:**
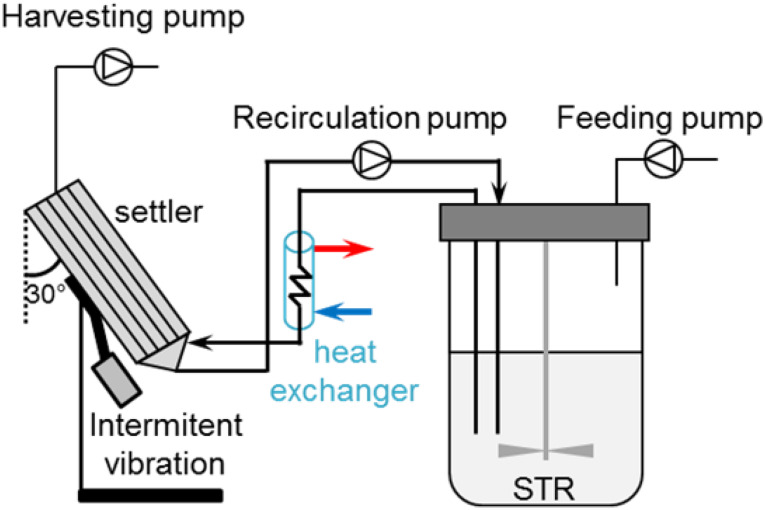
Perfusion cell culture set-up using an inclined settler. Cells are recirculated in a loop using a peristaltic pump at a flow rate of 35 mL/min. At the top of the inclined settler, another peristaltic pump harvests cell-free medium. The addition of fresh medium through the feeding pump allows maintaining the working volume at steady state. Blue and red arrows indicate the flow direction (water recirculation) in the heat exchanger.

Inclined settlers (ISs) allow cell separation through sedimentation due to the gravitational field (Castilho, 2014). Lamella settlers (IS) were developed for an increased sedimentation area compared to vertical settlers (Batt et al., 1990). Lamellas refer to plates, which are placed inside the equipment. On the surface of these lamellas, the cells sediment and subsequently slide to the bottom of the settler (Castilho, 2014). The cells are continuously circulated through the inclined settler and pumped back into the bioreactor (Figure 1). Intermittent vibrations applied on the IS help in a faster sedimentation of cells at the lower part of the IS. One important advantage of these devices is the preferential removal of non-viable cells and debris due to the size difference compared to viable cells. This results in the retention of predominantly viable cells (Batt et al., 1990). A disadvantage of this system is the relatively long residence time of cells in the IS (non-controlled environment; Voisard et al., 2003). To minimize side effects, cells that exit the bioreactor are cooled down in a heat exchanger before entering the IS in order to slow down the cell metabolism and increase sedimentation efficiency (Shen and Yanagimachi, 2011). ISs are simple and robust devices, which are successfully used for production of recombinant blood factors (Vogel et al., 2012), and in the seed train of fed-batch processes (Berrios et al., 2011) at scales up to 3000 L/day (Pohlscheidt et al., 2013). Perfusion operation for the production of biologicals using these devices can last up to 3–5 months (Henzler, 2012).

Here, we present the use of an IS for continuous harvesting of IAV. AGE1.CR.pIX cells were cultivated to high cell concentrations (>50 × 10^6^ cells/mL) in a stirred-tank bioreactor in perfusion mode. As a control, a perfusion cultivation was carried out at a cell concentration of 25 × 10^6^ cells/mL using an ATF2 system. Imaging flow cytometry was used to monitor the viral infection dynamics in the bioreactor. We show that the dynamics of infection and the IAV yields were strongly affected by the amount and timing of trypsin addition especially when using the inclined settler. The temperature of the heat exchanger (required for inclined settler operation) was shown to be a crucial parameter to obtain low cell population doubling times (t_d_) before infection. The effect of “cooling cells” (in the recirculation loop) on cell growth and virus production was studied in more detail in a cultivation using the same heat exchanger but with an acoustic settler as a control. For both inclined and acoustic settler, virus production was improved when using the heat exchanger. In perfusion cultivations with the IS, direct virus harvesting resulted in a maximum cell-specific virus yield (CSVY) of 3474 virions/cell. This corresponded to a 4.7-fold increase compared to an ATF cultivation performed as a control. With a volumetric virus productivity of 1.2 × 10^12^ virions/L/day, IS runs were 2.2-fold more productive than the cultivation with the ATF system.

## Materials and Methods

### Cell Line and Cultivation Conditions

The avian suspension cell line AGE1.CR.pIX (ProBioGen AG, Germany) was cultivated in a chemically-defined medium (CD-U3, ProBioGen AG, produced by Merck, Germany) supplemented with 10 ng/L LONG R^3^ IGF-I (Sigma), 2 mM L-glutamine (Sigma), and 2 mM L-alanine (Sigma). Baffled shake flasks were incubated at 37°C, 5% CO_2_ and 185 rpm. Shake flasks with 50 mL working volume (V_w_; VWR, # 89095–262) and 100–200 mL V_w_ (VWR, # 89095–270) were used for cell passaging and inoculum propagation, respectively.

### Virus and Infection Conditions

Human influenza A/PR/8/34 H1N1 virus seed produced using MCDK cells (Robert Koch Institute, Amp. 3138, TCID_50_ of 1.2 × 10^8^ virions/mL or 9.9 × 10^7^ virions/mL) was used in all experiments. After a complete medium exchange (performed by increasing the perfusion rate 2–3 h before infection), cells were infected with an MOI of 10^–5^ infectious virions/cell. At time of infection (TOI), porcine trypsin (Gibco, # 27250–018) was added from a stock solution (5000 U/mL in PBS). The detailed process parameters including the strategy of trypsin addition is shown in Table 1.

**TABLE 1:** Process parameters for bioreactor cultivations in perfusion mode during the infection phase with an inclined settler (IS), an acoustic settler (AS; control runs), and an ATF system (control run).

	Trypsin (U/mL)			Bioreactor working volume (mL)	T cooling water^d^ (°C)
	1st dose^a^	2nd dose^b^	Feed^c^		
IS3	38	38.0	–	650	27
IS4	12.5	15.0	–	650	27
IS5	25	15.0	–	650	27
IS6	12.5	–	2	650	27
AS1^*e*^	12.5	15.0	–	650	–
AS2^*e*^	12.5	12.5	–	650	27
ATF	12.5	20.0	–	800	–

*^a^1st dose addition at time of infection. ^b^2nd dose addition at 12–18 h post infection. ^c^Trypsin added in the feed medium (instead of adding a 2nd dose). ^d^Corresponds to the temperature at the inlet stream of the heat exchanger for an inclined settler or an acoustic settler (see Figure 1). ^*e*^Optimum process parameters were determined following a previous study (Gränicher et al., 2020).*

### Perfusion Bioreactor Cultivations

A 1 L stirred-tank bioreactor (STR) Biostat B Plus (Sartorius Stedim, Germany) coupled to an IS CS10 (Biotechnology Solutions, United States) was used for perfusion cultivations. Cells were inoculated at 1.0–1.2 × 10^6^ cells/mL in 950 mL V_w_. Settings for cultivations were pH 7.2, dissolved oxygen (DO) 40% air saturation and 120–180 rpm (pitched blade impeller). DO and pH were controlled by sparging of O_2_ and CO_2_, respectively. The IS was operated at a recirculation rate of 35 mL/min, intermittent vibration (15 s on, 10 min off) and with 30° angle, as described previously by Coronel et al. (2019b). Water at different temperatures was recirculated in the heat exchanger (between 20°C and 27°C). When the perfusion was started, the bioreactor V_w_ was decreased to 650 mL due to sampling and the dead volume in the IS (about 275 mL).

For the experiments using the acoustic settler (AS; 10 L acoustic chamber model, 20 mL V_w_, SonoSep Technologies), an acoustic power of 3 W and 2.1 MHz frequency was applied to all runs (Gränicher et al., 2020). Cells were inoculated at 1.2 × 10^6^ cells/mL in 650 mL V_w_. Through a pumping loop at a flow rate of 5 day^–1^, the cells from the bioreactor were circulated in the acoustic chamber (chamber volume of 20 mL). An additional loop with a heat exchanger was connected to the bioreactor for run AS2, similarly to the IS (recirculation flow rate of 35 mL/min), and cooled using a water recirculation system at 27°C (see Table 1).

For the membrane-based perfusion, an ATF2 system with C24U-v2 controller (Repligen, Waltham, Massachusetts, United States) and a 0.2 μm PES hollow-fiber membrane (Repligen, S02-P20U-10-N) was used. Cells were inoculated at 1.2 × 10^6^ cells/mL in 800 mL V_w_. The approximate volume exchanged with the ATF2 system was 100 mL. The exchange flow rate was set to 0.8 L/min.

For all cultivations, the perfusion was started before a minimum glucose concentration (determined as the limiting nutrient) of 17 mM was reached (Vazquez-Ramirez et al., 2018). The harvest flow rate was adjusted during the cell growth phase (before virus infection) to maintain a cell-specific perfusion rate (CSPR) of 0.06 nL/cell/day, previously established for this cell line (Vazquez-Ramirez et al., 2018), in order to keep a constant environment with no nutrient limitation.

For bioreactor cultivations with the IS, the acoustic settler or the ATF system, trypsin was added at TOI to facilitate IAV propagation (see Table 1). The ATF, IS4, IS5, AS1, and AS2 runs followed the same trypsin addition strategy, based on the cell concentration in the bioreactor (0.5 trypsin units per 10^6^ cells/mL). During the virus production phase, a second trypsin addition was done at 12–18 h post infection (hpi). In one run, trypsin was alternatively added to the feed medium. After infection, perfusion was interrupted for approximately 1 h to allow for virus entry. In the first experiment, the CSPR was maintained after infection, similarly to previous studies (Genzel et al., 2014; Coronel et al., 2019a), which resulted in perfusion rates of 0.6–2.0 day^–1^ (IS1) in this case. In the following cultivations, a constant perfusion rate of 2 day^–1^ was used after infection to allow for a shorter residence time of the virions inside the bioreactor.

The degree of cell separation can be determined by the retention efficiency (or cell separation efficiency). The reduced separation efficiency E′ (%; Eq. 1; Castilho and Medronho, 2002) is used for cell retention devices that operate with a fluid flow rate in the underflow such as gravity and acoustic settlers, sedimenting centrifuges and hydrocyclones. The E′ considers only those particles (cells) in the underflow, which were separated due to the capacity of the perfusion device, but not cells that were carried along in the stream due to drag force. In Eq. 1, X_o_ (cells/mL) is the viable cell concentration in the overflow stream (i.e., in the harvest) and X (cells/mL) is the viable cell concentration in the inlet (or feed stream), which can be assumed equal to the concentration measured in the bioreactor.

(1) $${\text{E}}^{\prime }=\,1-\,\frac{{\text{X}}_{\text{o}}}{\text{X}}$$

### Analytics

#### Cell Counting and Metabolites Measurement

Viable cell concentration and cell viability were determined using a Vi-CELL^®^ XR (Beckman Coulter, Brea, California, United States) as previously described (Lohr, 2014). Concentrations of glucose, lactate, glutamine, and ammonium (NH_4_^+^) were determined using a Bioprofile 100 Plus (Nova Biomedical, Waltham, Massachusetts, United States). All cell culture supernatants with virus were heat-inactivated (3 min, 80°C) before measurements (Genzel and Reichl, 2007).

#### Virus Titration

The concentration of infectious virions was determined using a TCID_50_ assay (infectious virions/mL), with a dilution error equal to ± 0.3 log_10_(infectious virions/mL; Genzel and Reichl, 2007). Hemagglutination assay was used to determine the HA titer in log_10_(HA units/100 μL) with a standard deviation of ±0.081 log_10_(HA units/100 μL; Kalbfuss et al., 2008). The concentration of virions (C_vir_, virions/mL) was estimated based on HA titer and erythrocyte concentration used in HA assay (2 × 10^7^ cells/mL; Eq. 2), assuming binding of one virion per erythrocyte. Based on the standard deviation of the HA assay, the error of C_vir_ is equal to 20.5% for the upper value and 17.0% for the lower value.

(2) $${\text{C}}_{\mathrm{vir}}\,=\,2\,\times \,10^{7}\times \,10^{\log_{10}\left(\mathrm{HA}\,\mathrm{units}/100\,\mu \,L\right)}$$

#### Imaging Flow Cytometry

Imaging flow cytometry was used to determine the fraction of cells infected with IAV. Samples were processed as described previously (Frensing et al., 2016; Coronel et al., 2019a). Therefore, samples containing 2 × 10^6^ cells were collected during virus production phase. Following fixation with paraformaldehyde, samples were stored in 70% ethanol at −20°C. Antibody staining for viral nucleoprotein (NP) combined with nuclear staining using DAPI were done according to Coronel et al. (2019a), adapted from Frensing et al. (2016).

In brief, the main steps were blocking at 37°C for 30 min; incubation with monoclonal mouse anti-NP antibody mAb61A5 [from F. Momose, (Frensing et al., 2016)] diluted 1:500; incubation with AF 647-conjugated polyclonal goat anti-mouse antibody (Life Technologies, A21235) diluted 1:500; addition of DAPI (approximately 5 μg/mL). Washing and dilution of antibodies were done using FACS buffer [PBS, 2% (w/v) glycine, and 0.1% (w/v) bovine serum albumin (BSA)]. Blocking was done in PBS, 1% (w/v) BSA. The incubation conditions for both antibodies were: 37°C, 60 min, in the dark.

Using an ImageStream^®^X Mark II Imaging Flow Cytometer (Amnis, Luminex, Austin, Texas, United States), 10,000 events (single cells) were collected. Acquisition was done using brightfield, and 375/642 nm excitation. The percentage of infected cells (NP positive) was determined using IDEAS software (version 6.2). Samples of cells from non-infected shake flasks with high cell viability (external control) and samples from 0 hpi in perfusion bioreactors (internal control) were used as negative controls.

#### Yield and Titer Calculations

The CSVY (virions/cell) was calculated as the ratio of the maximum total number of virions produced (Vir_tot, max_, virions), the maximum concentration of viable cells post infection (X_v,max_, cells/mL), and the corresponding bioreactor V_w_ (L; Eq. 3). The working volume in this case referred to the volume in the bioreactor and recirculation loop (inclined settler) or exchanged volume ATF. For perfusions using the acoustic settler, the V_w_ referred only to the bioreactor volume (the volume in the acoustic chamber is negligible). The volumetric virus productivity (P_v_, virions/L/day) was calculated from Vir_tot,max_, total spent medium V_tot_, L, and total process time (t_tot_, in days; Eq. 4). The Vir_tot_ (total number of virions produced) in turn considered the concentration of virions in the bioreactor (C_vir,br_, virions/mL) with V_w_ and the concentration of virions (C_vir, h_, virions/mL) of the accumulated virus harvest volume (V_h_, L; Eq. 5). The Vir_tot, max_ value corresponds to the maximum value of Vir_tot_.

Note: Although most virions were retained in the bioreactor in ATF cultivations, the concentration of virions in the permeate (C_vir, h_) was also taken into account in these calculations. In previous studies using ATF systems, the virion concentration in the permeate was neglected; thus, the CSVY and P_v_ equations reported previously only considered the maximum virion concentration measured in the bioreactor V_w_ (Nikolay et al., 2018; Gränicher et al., 2019; Vazquez-Ramirez et al., 2019; Coronel et al., 2019a).

(3) $$\mathrm{CSVY}=\frac{{\mathrm{Vir}}_{\mathrm{tot},\max}}{X_{v,\max}\times V_{w}}$$

(4) $$P_{v}=\frac{{\mathrm{Vir}}_{\mathrm{tot},\max}}{V_{\mathrm{tot}}\times t_{\mathrm{tot}}}$$

(5) $${\mathrm{Vir}}_{\mathrm{tot}}=C_{\mathrm{vir},\mathrm{br}}\times V_{w}+\sum C_{\mathrm{vir},h}\times V_{h}$$

To compare the Vir_tot, max_ values calculated from different bioreactor cultivations with changes in the V_w_, all runs were normalized to 650 mL V_w_. For a comparison between the different CSVY, P_v_, and Vir_tot_ values, the relative standard deviation of the titration assay only (section “Virus titration”) was considered, resulting in an error equal for all three calculations to 20.5% for the upper value and 17.0% for the lower value.

To evaluate the metabolic status of cells, the cell-specific glucose uptake rate (q_glc_) and the lactate yield based on glucose consumption (Y_lac/__glc_) were calculated as described previously (Gränicher et al., 2019).

## Results

To allow an efficient IAV production in perfusion mode using an IS, investigations started with the characterization of cell growth before infection. Once a short t_d_ was reached for AGE1.CR.pIX cells, IAV production was evaluated by calculating Vir_tot_, CSVY, and P_v_. Results were compared to a virus production process also operated in perfusion mode but coupled to an ATF system (see section “Perfusion bioreactor cultivations”). However, the latter did not allow for an efficient continuous virus harvesting through the hollow-fiber membrane. Two cell concentrations at time of infection and different trypsin addition strategies were tested with the IS. Furthermore, the temperature setting of the heat exchanger seemed to have an impact on cell growth and virus production. Therefore, this was further studied by using another cell retention device, an acoustic settler, equally enabling continuous virus harvesting. In contrast to an IS, a setting with and without heat exchanger was possible for the acoustic settler.

### Conditions for Efficient Cell Growth in Perfusion Mode Using an Inclined Settler

The t_d_ in perfusion was evaluated for different recirculation water temperatures of the heat exchanger of the inclined settler device (illustrated in Figure 1).

**FIGURE 2:**
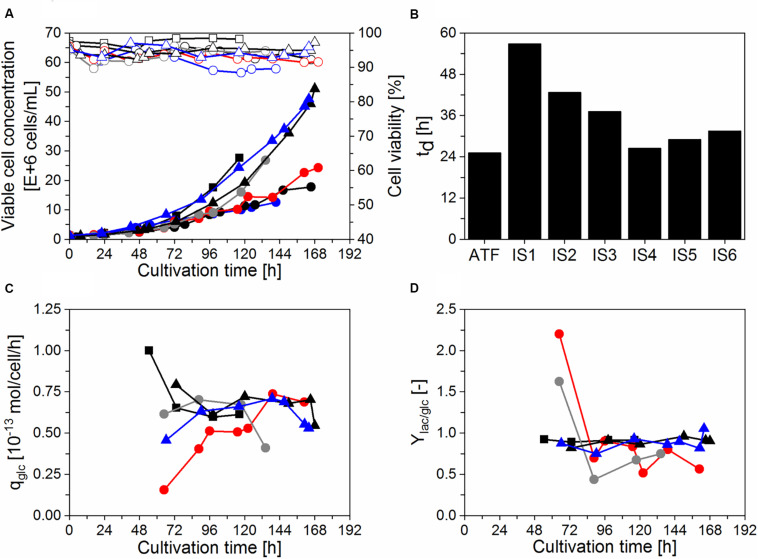
Growth and metabolism of AGE1.CR.pIX cells in perfusion mode using a stirred-tank bioreactor coupled to an inclined settler or an ATF system. Cultivations with an inclined settler (IS): IS1 (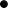), IS2 (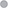), IS3 (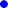), IS4 (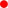), IS5 (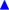), and IS6 (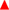). Cultivation with the ATF system (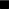). **(A)** Viable cell concentration (filled symbols) and cell viability (empty symbols). **(B)** Doubling time (t_d_) during the cell growth phase. **(C)** Cell-specific glucose consumption rate (q_glc_) during perfusion (after 48 h). **(D)** Lactate yield based on glucose consumption (Y_lac/__glc_) during perfusion (after 48 h).

Initial cultivations were done using a thermostatic bath to cool the water in the heat exchanger, that is commonly used during IS operation in biopharmaceutical production using other cell lines (e.g., CHO cells; Choo et al., 2007; Pohlscheidt et al., 2013; Coronel et al., 2019b). In our case, AGE1.CR.pIX cells did not efficiently grow (t_d_ > 48 h) with set-points varying between 20–22°C (IS1, Figures 2A,B), so higher set-points were used during the cell growth phase for run IS2 (25–27°C; Figures 2A,B). A temperature of 27°C was also reached in a process with simple water recirculation without cooling. This enabled successful cell growth at high viabilities (>92%) in four follow-up bioreactor experiments (IS3–6, Figures 2A,B) and were used for infection studies with IAV (section “Influenza A virus production in perfusion cultures”). Growth up to 25 × 10^6^ cells/mL (IS3, IS4) or 50 × 10^6^ cells/mL (IS5, IS6) before addition of the virus seed. In the cultivation IS3 with settler operation at RT, t_d_ was improved (37 h) compared to IS1–2. In the cultivations IS1, IS2, and IS3, recirculation was started 48 h after inoculation, corresponding to the middle of the exponential cell growth phase. In the following cultivations (IS4, IS5, and IS6), recirculation was started at time of inoculation. This resulted in a further improvement of t_d_ in the range 26–32 h. The results indicated that both the temperature of the heat exchanger of the inclined settler and the time point of starting the recirculation have an impact on cell growth. In a cultivation using an ATF system performed as a control, a maximum concentration of about 25 × 10^6^ cells/mL and a slightly lower t_d_ (25 h) were achieved (Figures 2A,B).

For all the perfusion runs, the perfusion flow rate was adjusted daily to maintain the desired CSPR during the cell growth phase as described in section “Perfusion bioreactor cultivations.” Under these conditions, glucose concentration always exceeded 2 g/L (data not shown). In addition, q_glc_ and Y_lac/__glc_ were analyzed for the successful runs (IS3–6 and ATF) to assess if the use of a recirculating loop coupled to a heat exchanger has an influence on cellular metabolism. As before, a cultivation with ATF-based perfusion served as a control. Similar q_glc_ and Y_lac/__glc_ were observed for both systems (Figures 2C,D). For IS3, a lower q_glc_ was observed between 48 h and 120 h, which was likely associated with the start of recirculation on day 2 leading to a slowdown of cell growth and metabolism. For IS4, a lower Y_lac/__glc_ was observed between 72 h and 96 h.

### Influenza A Virus Production in Perfusion Cultures

**FIGURE 3:**
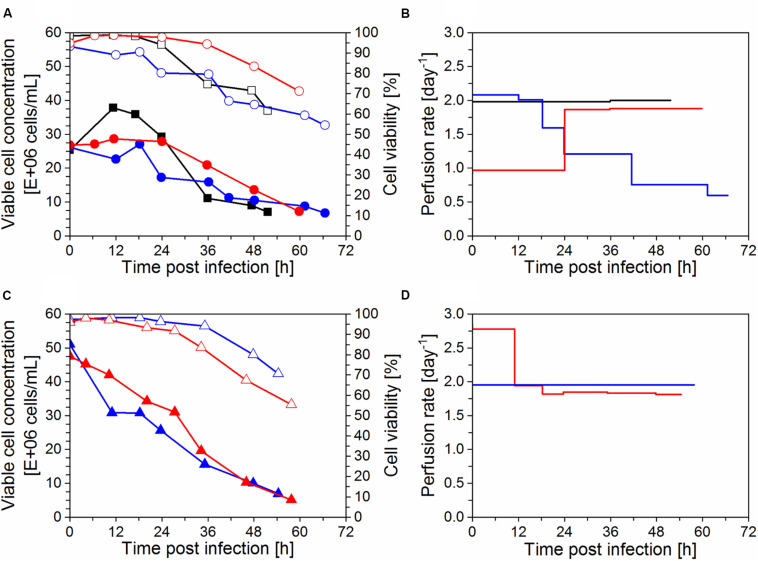
Production of influenza A virus in perfusion mode using AGE1.CR.pIX cells (time of infection *t* = 0 h). Cultivations in stirred-tank bioreactor with an inclined settler (IS) IS3 (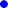), IS4 (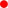), IS5 (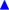), and IS6 (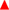) plus one control run with an ATF system (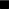) were carried out. **(A, B)** Cells were infected at 25 × 10^6^ cells/mL (IS3, IS4, and ATF) or **(C, D)** 50 × 10^6^ cells/mL (IS5, IS6). **(A, C)** Viable cell concentration (filled symbols) and cell viability (empty symbols) shown as average of analytical duplicates. **(B, D)** Perfusion rate in bioreactor working volume per day (day^–1^).

After infection, the bioreactor was operated at a constant perfusion rate of 2 day^–1^, except for IS3, which was operated CSPR-based during the entire cultivation period (Figure 3B). When cells are infected with IAV at low MOIs (10^–3^–10^–5^ infectious virions/cell), they can typically continue to grow for 12–24 hpi, until the majority of the cells is infected and the virus titer starts to increase strongly. To avoid substrate limitations, the CSPR was maintained during the initial virus production phase for cultivation IS5 infected at 50 × 10^6^ cells/mL (Figure 3D). However, no glucose depletion was observed in a control experiment (IS6) infected at the same cell concentration (data not shown).

After infection with IAV, viable cell concentrations varied according to infection conditions and perfusion system used (described in sections “Virus and infection conditions” and “Perfusion bioreactor cultivations”). For the cultivations infected at 25 × 10^6^ cells/mL (Figure 3A), the cell concentration was maintained after infection in the IS cultivations whereas cell growth continued for about 12 hpi in the ATF culture. A comparison between IS3 (infected with 38 trypsin U/mL) and IS4 (12.5 trypsin U/mL; Table 1) suggests that a lower trypsin activity (IS4) allowed for a better cell growth after infection. Nevertheless, even though the same trypsin activity was used in experiments IS4 and ATF (12.5 U/mL; Table 1), different cell growth profiles were obtained (Figure 3A). The concentration reached 38 × 10^6^ cells/mL for the ATF culture after infection, while the concentration did not exceed 30 × 10^6^ cells/mL for the runs using the IS. These results may suggest that infected cells in medium containing trypsin are less robust and more affected by ISs than ATF systems due to higher shear forces in the former (also see t_d_ and cell concentrations, Figures 2A,B). In particular, the use of the peristaltic pump in the recirculation loop may result in increased cell damage using ISs. In addition, cooling to 27°C might play a role in IS cultivations.

For infection at 50 × 10^6^ cells/mL (Figure 3C), trypsin activities between 12.5 and 25 U/mL were employed (Table 1). In addition, one of the runs (IS6) was operated with trypsin supplementation in the feed medium (2 U/mL) instead of adding a second dose. Interestingly, a rapid decrease in viable cell concentration occurred soon after infection in the cultivations IS5 and IS6. This was in clear contrast to the behavior obtained in those infected at 25 × 10^6^ cells/mL (IS3, IS4; Figure 3A). The effect was more pronounced for IS5 (25 U/mL) compared to IS6 (12.5 U/mL). This behavior was also observed in pseudo-perfusion experiments in spin tubes previously carried out to select the best infection conditions using 12.5–25 U/mL of trypsin (data not shown).

**FIGURE 4:**
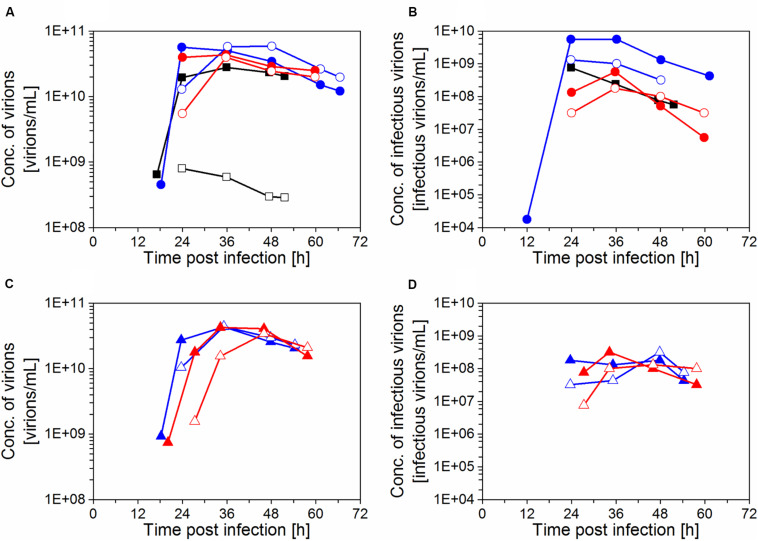
Influenza A virus production in perfusion cultivations of AGE1.CR.pIX cells with inclined settler (IS) IS3 (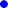), IS4 (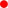), IS5 (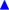), IS6 (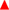), and ATF system (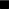). **(A, C)** Concentration of virions in the bioreactor and in the harvest, based on HA titer; **(B, D)** concentration of infectious virions, based on TCID_50_. The samples were taken from the bioreactor (filled symbols) and the harvest (empty symbols).

Maximum C_vir, br_ and C_vir, h_ values in the range of 3.4–5.9 × 10^10^ virions/mL were obtained for cultures with the inclined settler (IS3–IS6), whereas the highest titer with the ATF system was slightly lower with 2.8 × 10^10^ virions/mL (Figures 4A,C). However, the increase of C_vir_ was in the range of the error of the titration assay (section “Virus titration”). The virus titers measured in the harvest line of the inclined settler followed a profile very similar to that measured in the bioreactor, demonstrating efficient continuous harvesting with this retention device. The small delay in achieving the maximum titer in the harvest compared to the bioreactor could be related to the dead volume of the inclined settler unit. In the experiment with the ATF system, very low virus titers were measured in the harvest, corroborating previous findings regarding membrane blocking (Gränicher et al., 2019; Vazquez-Ramirez et al., 2019).

Maximum TCID_50_ titers achieved 36–48 hpi were in the range 1.0–5.6 × 10^8^ infectious virions/mL in IS cultivations and 7.6 × 10^8^ infectious virions/mL with ATF system at 24 hpi (Figures 4B,D). For the ATF-based cultivation, maximum TCID_50_ values were achieved earlier, which was most likely due to faster virus accumulation in the bioreactor after membrane blocking and, eventually, also due to the absence of a cooling system. For the IS experiments, viruses produced in the bioreactor were constantly harvested via the permeate. However, highest infectious titers for the IS cultivations were almost always measured for samples taken from the bioreactor but not from the harvest. This finding suggests that the infectivity of virions decreased during the passage through the heat exchanger and the settler device. Nevertheless, overall, a total of 2.5–6.1 × 10^11^ infectious virions was produced (Eq. 3), similarly to the ATF culture (5.0 × 10^11^ infectious virions).

The maximum total number of virions produced based on the HA titer (Eq. 3) for IS3–6 was in the range of 5.4–6.5 × 10^13^ virions, which represents a 3.2-fold increase compared to the ATF culture (Table 2). Very high CSVYs were obtained with the inclined settler, providing a 4.7-fold (IS3, IS4) or 2.6-fold (IS5, IS6) increase compared to the ATF culture (control). Since the cell growth phase was usually extended with the inclined settler, the increase in P_v_ was of 2.2-fold (IS3, IS4), and 1.4-fold (IS5, IS6) compared to the ATF culture (Table 2). Except for the P_v_ of IS5 and IS6, the increase of the Vir_tot, max_, CSVY, and P_v_ between the ATF and IS3–6 exceeded the error of the titration assay (section “Yield and titer calculations”).

**TABLE 2:** Concentration of AGE1.CR.pIX cells and influenza A virus yields for perfusion runs using the inclined settler (IS) or the ATF system.

Run	X_v,_ viab.^a^	Vir_tot, max_^b^	CSVY^c^	P_v_^d^
	(cell/mL,%)	(10^13^ virions)	(virions/cell)	(×10^12^ v/L/d)
IS3	24 × 10^6^, 92	5.7	3259	1.18
IS4	27 × 10^6^, 95	6.5	3474	1.23
ATF	25 × 10^6^, 99	1.9	723	0.55
IS5	52 × 10^6^, 97	6.5	1953	0.83
IS6	48 × 10^6^, 96	5.4	1753	0.66

*^a^Viable cell concentration (X_v_) and cell viability (viab.) at time of infection. ^b^Maximum total number of virions produced (Vir_tot, max_), normalized to a bioreactor volume of 650 mL (see section “Yield and titer calculations”). ^c^Cell-specific virus yield (CSVY). ^d^Volumetric virus productivity (P_v_).*

**FIGURE 5:**
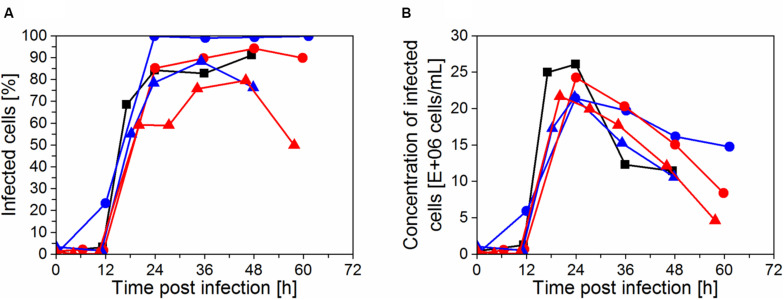
Progression of infection of cells with influenza A virus in perfusion cultivations determined by imaging flow cytometry. **(A)** Fraction of infected cells positive for virus nucleoprotein and **(B)** concentration of infected cells in the bioreactor, calculated from the measured total cell concentration and the fraction of infected cells. Runs: IS3 (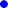), IS4 (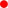), IS5 (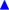), IS6 (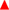), and ATF (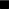).

The progression of virus infection in the cultivations was determined by flow cytometry as the fraction of infected cells (Figure 5A). The highest trypsin activity (1.5 × 10^–6^ U/cell or 38 U/mL) in the IS3 run led to a complete infection of the cell population at 24 hpi. For the runs IS4, IS5, and ATF that were infected using a lower dose of trypsin (0.5 × 10^–6^ U/cell or 12.5–25 U/mL), only 80–85% of cells were infected at 24 hpi and the peak infectivity was delayed to 36–48 hpi, corresponding to 90–95% of infected cells. Finally, when trypsin activity at TOI was further reduced in the cultivation IS6 (0.25 × 10^–6^ U/cell or 12.5 U/mL) and trypsin was fed in the medium during virus production phase, the percentage of infected cells at 24 hpi was considerably lower (60%, Figure 5A). A maximum of 80% was obtained at 48 hpi. However, at this time point, the virus production phase was nearly completed. Therefore, the concentration of infected cells was low (Figure 5B). Clearly, for maximum virus production, the majority of the cells should be infected within 24 h after addition of the virus seed when viability is also highest.

Although runs IS5 and IS6 were infected at 50 × 10^6^ cells/mL, the actual number of infected cells at 24 hpi was approximately the same compared to the cultures infected at 25 × 10^6^ cells/mL (IS3, IS4, and ATF; Figure 5B). Therefore, a significant fraction of the cells present at the time of infection in IS5 and IS6 was not infected and/or a fraction of infected cells likely died before replicating and releasing progeny virions (which usually starts at about 6–8 hpi). Accordingly, virus production was similar in the four experiments with the IS (Figures 4A,C). Since the CSVY calculation considers the maximum cell concentration from time of infection (TOI) onwards, lower CSVYs were obtained in the case of experiments IS5 and IS6 (Table 2).

### Influence of the Heat Exchanger on Virus Production

In order to evaluate the impact of the heat exchanger and cooling during perfusion and continuous virus harvesting, an acoustic settler was used in a setting similar to a previous study (Gränicher et al., 2020). This cell retention device also enables continuous virus harvesting, but does not require the use of a heat exchanger.

**FIGURE 6:**
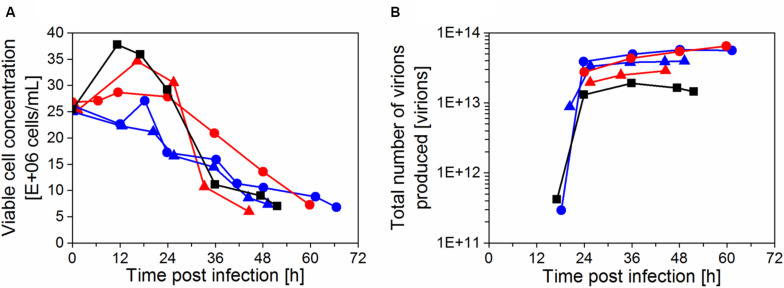
**(A)** Cell growth and **(B)** total number of virions produced (Vir_tot_, based on HA titer) after infection using an acoustic settler without heat exchanger (AS1, 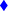) or with heat exchanger (AS2, 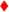), compared to runs IS3 (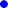), IS4 (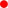), and ATF (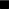). The total number of virions produced was normalized to a bioreactor working volume of 650 mL (see section “Yield and titer calculations”).

Under the same infection conditions and same perfusion strategy (sections “Virus and infection conditions” and “Perfusion bioreactor cultivations”), two perfusion runs were performed using the acoustic settler either without (AS1) or with (AS2) heat exchanger, and compared to cultures with an inclined settler (IS3–4) and the ATF system (Figure 6).

As observed previously (section “Influenza A virus production in perfusion cultures”), the maximum viable cell concentration after infection decreased from 35 × 10^6^ cells/mL (AS1) to 25 × 10^6^ cells/mL (AS2) for perfusion systems using a heat exchanger (Figure 6A). Similar cell growth and virus release profiles were observed for the cultivations with a heat exchanger, either with the acoustic settler (AS2) or the inclined settler (IS3, IS4; Figures 6A,B). Based on HA titers, a higher total number of virions were produced in these runs (AS2, IS3, and IS4) compared to cultures using an acoustic settler without heat exchanger (AS1) and the ATF system. The difference in virus release was detected at the limit of the titration error. Yet, the maximum total number of virions produced was only slightly higher for IS3 and IS4 (5.7–6.5 × 10^13^ virions) compared to cultures using an acoustic settler with heat exchanger (AS2, 4.0 × 10^13^ virions; Figure 6B).

## Discussion

### Growth of AGE1.CR.pIX Cells in Perfusion Mode Using an Inclined Settler

The perfusion cultivations performed in a STR coupled to the IS (CS-10) yielded cell concentrations up to 50 × 10^6^ cells/mL, with viabilities above 92% (Figure 2). The experimental set-up involved cell recirculation with a peristaltic pump operated at 27°C. Cell doubling times between 26–32 h (IS4, IS5, and IS6) were obtained, when recirculation was started directly after inoculation. This is at the lower range of results previously reported for perfusion cultivations using AGE1.CR cells (t_d_ 30–44 h; Genzel et al., 2014) and AGE1.CR.pIX cells (t_d_ 29–40 h; Vazquez-Ramirez et al., 2019) in STR with ATF systems, or cultivations with AGE1.CR.pIX cells (t_d_ 26–43 h) in an orbital-shaken bioreactor with ATF and TFF systems (Coronel et al., 2019a).

Although other cell lines such as CHO cells were reported to grow efficiently when cooling the inclined settler to temperatures lower than 22°C (Choo et al., 2007; Pohlscheidt et al., 2013; Coronel et al., 2019b), the AGE1.CR.pIX cells seemed to be sensitive to low temperatures in the recirculation loop. A previous case study showed through orthogonal partial least square multivariate analysis that the temperature in the inclined settler is one of the most important factor for the productivity variability (Shimoni et al., 2018). It seems that in our case, temperature has also to be selected carefully to increase process performance.

Cooling of the IS is necessary as it enables to slow down cell metabolism, to maintain high cell viabilities and a high cell retention efficiency E’. In the present study, E’ was maintained between 96% and 99% during the cell growth phase for IS3–6, showing no further need to cool the cells in the recirculation loop. As reviewed by Castilho and Medronho (2002), the separation efficiency is determined by the terminal settling velocity of particles in a laminar flow (Stokes’ law). Among other factors, it is related to the cell diameter. With the onset of apoptosis and cell death after infection, the average diameter of the cell population typically decreases over the course of virus production phase. Hence, a gradual drop in the separation efficiency can occur, especially at late stages of infection. For the inclined settler cultivations with infection at low MOI (IS3–IS6), the optimum harvest time regarding maximum total virus production was around 60 hpi. E’ remained reasonable high (>85%) until the processes were ended. Minimum E’ values measured at 54–60 hpi were 93% (IS3), 96% (IS4), 94% (IS5), and 85% (IS6). Therefore, the reduction of E’ during virus production was not critical for our application.

In one scouting experiment maintained for a longer period after infection of AGE1.CR.pIX cells with IAV, E’ dropped to 86–50% at 67–92 hpi. At a later stage of infection (116 hpi), nearly the same cell concentration was measured in the bioreactor and in the harvest (E’ ≈ 0). In this example, the average cell diameter was 14.0 μm during the cell growth phase and 10.8 μm at the end of the run. Accordingly, for processes with lytic viruses and a fast decrease in separation efficiency, continuous virus harvesting might be problematic using inclined settlers.

Another factor with a high impact on cell retention efficiency for operation with an inclined settler is the harvest flow rate (Shen and Yanagimachi, 2011). Therefore, equipment with a suitable capacity should be selected. The model CS-10 used in our work, which has a total area of 0.046 m^2^, was designed for operation at flow rates up to 8 L/day. In our experiments, the cultivations were carried out using 2–3 day^–1^ (1.30–1.95 L/day), thus enabling high retention efficiencies, as previously mentioned. At large scale, 99% efficiency was reported for a biopharmaceutical process using perfusion rates over 2000 L/day for steady state at 20 × 10^6^ cells/mL or higher (Vogel et al., 2012), demonstrating that this perfusion device can be successfully scaled up with high E’.

For comparable t_d_ values, the cell-specific glucose uptake rate q_glc_ and the lactate yield based on glucose consumption Y_lac/__glc_ were in a similar range for cultivations with the inclined settler compared to the ATF culture (IS4–6 and ATF, Figures 2C,D). Therefore, cell recirculation in the inclined settler at 35 mL/min at 27°C seems not to influence significantly AGE1.CR.pIX cell growth and metabolism compared to an ATF cultivation. The conserved q_glc_ further suggests that the cell-specific perfusion rate adjusted to 0.06 nL/cell/day [based on glucose consumption as previously described (Vazquez-Ramirez et al., 2018; Vazquez-Ramirez et al., 2019)] was equally suitable for IS cultivations.

### Virus Production Using an Inclined Settler

To our knowledge, the described process using an IS is the first report on the use of this technology in virus vaccine production. High volumetric productivities and CSVYs were obtained.

Infections at 25 × 10^6^ cells/mL enabled very high CSVYs and P_v_s in perfusion mode with the IS (4.7-fold and 2.2-fold increase compared to the ATF control run; Table 2). For infections at 50 × 10^6^ cells/mL, the CSVY was increased by a factor of 2.6. Cultivations using AGE1.CR.pIX cells for the production of human influenza A/PR/8/34 H1N1 virus in batch mode in STR led to a CSVY of 1344 virions/cells (Vazquez-Ramirez et al., 2019). Cultivations in perfusion mode with ATF systems or hybrid fed-batch/perfusion processes for the production of the same IAV strain using the same cell line yielded 340–1300 virions/cell (Vazquez-Ramirez et al., 2019). Other ATF perfusion cultivations using the parental AGE1.CR cells resulted in yields of 518–1708 virions/cell (Genzel et al., 2014). The CSVY obtained in the present work for IS cultivations infected at 25 × 10^6^ cells/mL (3259–3474 virions/cells for IS3–4) clearly exceed those reported for batch or perfusion cultivations carried out in STRs. In terms of P_v_, virus production using an IS allowed a 1.9-fold increase compared to a fed-batch process using the same cell line and virus strain (Vazquez-Ramirez et al., 2019), showing the potential of IAV production in perfusion mode using an IS. The obtained CSVY (1753–1953 virions/mL) with the IS at a cell concentration of 50 × 10^6^ cells/mL were also higher than in previous studies for cultivations performed in perfusion mode (Genzel et al., 2014) using an ATF system producing the same virus strain with a very similar cell line (AGE1.CR cell line; 50 × 10^6^ cells/mL at TOI; CSVY 1266 virions/cell). Further comparisons with other processes reported in literature would be difficult as host cells, media, virus strain and virus titration assays differ (Gränicher et al., 2019).

We believe that the high values obtained for CSVY in perfusion mode using an IS were to a certain degree related to the continuous virus harvesting strategy. In addition, high virus titers and yields were also mediated by variation in culture temperature between the bioreactor and IS. Even though the temperature in the bioreactor was maintained constant at 37°C, the cell suspension was cooled in the heat exchanger but the IS kept at RT. Therefore, the cells were subjected to a temperature gradient of about 10°C. Petiot et al. (2011) investigated the stability and yield of IAV produced in HEK293 cells at different temperatures (37°C and 35°C). In this study, virus degradation was less pronounced for processes operated at 35°C with storage of the supernatant at 2–8°C. Consequently, virus titers and final yields were higher under these conditions. Lower temperatures of 32–35°C were also reported for production of IAV in various cell lines including MDCK and Vero (Genzel and Reichl, 2009), suggesting that production may benefit from lower temperatures. The use of the heat exchanger induced a very different cell growth profile after infection not only for the IS but also the acoustic settler. In particular, for cultivations performed with a heat exchanger, the maximum cell concentration obtained after infection was reduced (compared to cultivations without heat exchanger, AS1, and ATF). This could be an advantage as virus production instead of cell growth seemed to be promoted under these conditions, in addition to reduced virus degradation. This is also supported by the results obtained for the cultivations with the acoustic settler with or without heat exchanger (section “Influence of the heat exchanger on virus production”).

Concerning infectious titers, no major differences were observed between ATF and IS cultivations. Maximum titers of 1.8–7.6 × 10^8^ infectious virions/mL were obtained. In addition, virus degradation was observed in the perfusion cultivations toward the end of the virus production phase regardless of the experimental conditions evaluated (Figure 4). One option for future studies with the focus to achieve higher concentrations of infectious particles (for life-attenuated vaccines) using an IS is to increase the perfusion rate. This way, the residence time of virions in the system is decreased, potentially reducing the degradation. Furthermore, it has to be taken into account that virions accumulating inside the bioreactor may be subjected to degradation not only by low temperature stability but also due to the release of cellular proteases at later stages of the infection phase (Gallo-Ramirez et al., 2015).

Recently, a single-use orbital-shaken bioreactor (OSB) was evaluated by our group for human influenza A/PR/8/34 H1N1 virus production with AGE1.CR.pIX cells up to 10 L V_w_. Yields of 1055–3487 virions/cell were obtained in perfusion mode with either ATF or TFF systems (Coronel et al., 2019a). ISs are traditionally made of stainless steel (Pohlscheidt et al., 2013), however, novel single-use models of compact settlers are being produced (Kompala et al., 2018), which can be used for perfusion cultivations, besides harvesting and clarification. These devices were developed mainly for perfusion cultivations of small cells such as yeasts, to improve the cell retention efficiency and reduce the footprint of the equipment. The main difference is the conical shape of the external chamber and the high number of internal plates with an unusual helical arrangement (Freeman et al., 2017). Due to the many advantages of single-use technology (Galliher, 2017), it could be interesting to evaluate single-use ISs combined to single-use bioreactor systems, such as OSB, for virus production in future studies. Process parameters such as recirculation flow rate and the effect of the heat exchanger on cell retention efficiency and process productivity should then be re-evaluated.

Influence of trypsin activity on IAV production and cell metabolism after infection was observed in our conducted experiments. Trypsin activates influenza virus through cleavage of hemagglutinin (HA; Lazarowitz and Choppin, 1975) involved with attachment to cells and consequently with virus entry (Dou et al., 2018). The absence of this enzyme can lead to delayed virus propagation and reduced influenza virus yields (Seitz et al., 2012). In the present study, initial experiments were performed with infection at 25 × 10^6^ cells/mL using 0.5–1.5 × 10^–6^ U/cell (12.5–38.0 U/mL) of trypsin (IS3, IS4, and ATF; Table 1). Although different cell growth profiles post-infection were observed, similar virus production was achieved in IS3 and IS4 cultivations, which was significantly higher compared to the ATF cultivation (Table 2). Subsequently, an infection at 50 × 10^6^ cells/mL (IS5) was carried out using the lower limit previously tested in terms of trypsin activity per cell, that is 0.5 × 10^–6^ U/cell (25 U/mL). This corresponded to an activity two times higher in terms of unit per volume compared to IS4 and ATF (12.5 U/mL). Interestingly, a drop in viable cell concentration was observed (Figure 3C). One hypothesis to explain this finding was that the trypsin activity may have a negative impact on cultures at high cell concentrations in perfusion mode with an IS. In the subsequent perfusion cultivation (IS6), a lower trypsin activity was used at time of infection, i.e., 0.25 × 10^–6^ U/cell (12.5 U/mL) and a feed medium with a low volumetric trypsin activity (2 U/mL) provided. Similarly to cultivation IS5, the viable cell concentration started to decrease for IS6 directly after infection (although this effect was less pronounced), leading to equivalent virus yields in the perfusion experiments for IS5 and IS6 (Table 2). Analysis of the progression of infection over time showed a lower percentage of infected cells in the cultivations infected at 50 × 10^6^ cells/mL compared to those at 25 × 10^6^ cells/mL (Figure 5).

Although cells are usually less robust after virus infection, previous perfusion cultivations with the ATF system using the same cell line and virus did not indicate a significant decrease in cell growth during early stages of infection (Genzel, 2015; Vazquez-Ramirez et al., 2019). In these studies, infection with IAV H1N1 took place at high concentrations at TOI (up to 50 × 10^6^ cells/mL), using an MOI 10^–3^ in the presence of 1 × 10^–6^ U/cell trypsin. Therefore, similar trypsin activities seem to have different effects on process performance for cultivations with an ATF system or an inclined settler.

One difference between both systems is that the bottom part of an IS is filled with a large number of cells, which have settled and are returned to the bioreactor in the underflow. In this region, limitations in the supply of oxygen or nutrients may occur. According to Shimoni et al. (2018), the cell concentration can be 3–5 × higher compared to that of the cell suspension in the upper part of the bioreactor. For infection studies with IAV, an increased oxygen demand after trypsin addition is usually observed (Coronel et al., 2019a). Accordingly, for the IS5–6 cultivation performed at a high viable cell concentration at TOI, oxygen depletion might have played a role in reducing virus titers. In addition, cells subjected to trypsin at high cell concentrations (50 × 10^6^ cells/mL), may also be more sensitive to the shear stress induced by the recirculation pump operated at 35 mL/min. Perfusion cultivations performed at 50 × 10^6^ cells/mL using a low shear recirculating pump (e.g., a Levitronix MagLev pump) with variation in trypsin activities and recirculation flow rates, could help to clarify this detail in a future study.

One limitation of our study is the lack of a cost analysis comparing both perfusion systems using SuperPro Designer^®^ software, for instance. So far, however, our focus was on feasibility and efficiency assessments of cultivations with an IS compared to ATF-based systems based on P_v_ and CSVY values. The higher P_v_ achieved for the IS indicates that a cost reduction could be feasible as less cell culture medium and time would be needed to obtain a similar volumetric virus productivity in perfusion mode.

In conclusion, the selection of cell retention devices was shown to be crucial for process performance in IAV vaccine production. For concentrations in the range of 24–27 × 10^6^ cells/mL, inclined settlers seem to be a good choice to achieve high CSVYs and volumetric productivities. Compared to the use of an ATF system, a 4.7-fold (CSVY) increase and a 2.2-fold (P_v_) increase, were obtained. In part, this increase seems to be related to a continuous virus harvesting regime and the use of a heat exchanger operated at 27°C promoting virus production while decreasing the risk of virus degradation. Especially for temperature labile viruses or for live vaccines that require optimization of infectious titers, the option to continuously harvest without the risk of membrane blockage could make the difference in process intensification, and render cell culture-based virus production a viable alternative to traditional manufacturing.

## Data Availability Statement

The datasets generated for this study are available on request to the corresponding author.

## Author Contributions

JC, GG, VS, TN, YG, and UR contributed to the conception and design of the study. JC and GG performed the experiments. JC, GG, and YG analyzed the data. JC and GG wrote the manuscript. All authors contributed to manuscript revision, read, and approved the submitted version.

## Conflict of Interest

VS is employed by the company ProBioGen AG providing the cell line for the study. VS is listed as an inventor on a patent that protects the cell line AGE1.CR.pIX (WO 2005/042728). JC, GG, VS, TN, YG, and UR declare that the research was conducted in the absence of any commercial or financial relationships that could be construed as a potential conflict of interest.
